# Risk Factors for COVID-19 in College Students Identified by Physical, Mental, and Social Health Reported During the Fall 2020 Semester: Observational Study Using the Roadmap App and Fitbit Wearable Sensors

**DOI:** 10.2196/34645

**Published:** 2022-02-10

**Authors:** Kristen N Gilley, Loubna Baroudi, Miao Yu, Izzy Gainsburg, Niyanth Reddy, Christina Bradley, Christine Cislo, Michelle Lois Rozwadowski, Caroline Ashley Clingan, Matthew Stephen DeMoss, Tracey Churay, Kira Birditt, Natalie Colabianchi, Mosharaf Chowdhury, Daniel Forger, Joel Gagnier, Ronald F Zernicke, Julia Lee Cunningham, Stephen M Cain, Muneesh Tewari, Sung Won Choi

**Affiliations:** 1 Department of Pediatrics University of Michigan Medical School Ann Arbor, MI United States; 2 Department of Mechanical Engineering University of Michigan Ann Arbor, MI United States; 3 Stephen M. Ross School of Business University of Michigan Ann Arbor, MI United States; 4 Institute for Social Research University of Michigan Ann Arbor, MI United States; 5 School of Kinesiology University of Michigan Ann Arbor, MI United States; 6 Department of Computer Science Engineering University of Michigan Ann Arbor, MI United States; 7 Department of Mathematics University of Michigan Ann Arbor, MI United States; 8 Department of Orthopedic Surgery University of Michigan Ann Arbor, MI United States; 9 Department of Epidemiology University of Michigan Ann Arbor, MI United States; 10 Exercise & Sport Science Initiative University of Michigan Ann Arbor, MI United States; 11 Department of Chemical and Biomedical Engineering West Virginia University Morgantown, WV United States; 12 Department of Internal Medicine University of Michigan Ann Arbor, MI United States; 13 Department of Biomedical Engineering University of Michigan Ann Arbor, MI United States; 14 Center for Computational Medicine and Bioinformatics University of Michigan Ann Arbor, MI United States; 15 Veterans Administration Ann Arbor Healthcare System Ann Arbor, MI United States

**Keywords:** mHealth, mobile health, college student, mental health, wearable devices, wearable, student, risk factor, risk, COVID-19, physical health, observational, crisis, self-report, outcome, physical activity, wellbeing, well-being

## Abstract

**Background:**

The COVID-19 pandemic triggered a seismic shift in education to web-based learning. With nearly 20 million students enrolled in colleges across the United States, the long-simmering mental health crisis in college students was likely further exacerbated by the pandemic.

**Objective:**

This study leveraged mobile health (mHealth) technology and sought to (1) characterize self-reported outcomes of physical, mental, and social health by COVID-19 status; (2) assess physical activity through consumer-grade wearable sensors (Fitbit); and (3) identify risk factors associated with COVID-19 positivity in a population of college students prior to release of the vaccine.

**Methods:**

After completing a baseline assessment (ie, at Time 0 [T0]) of demographics, mental, and social health constructs through the Roadmap 2.0 app, participants were instructed to use the app freely, wear the Fitbit, and complete subsequent assessments at T1, T2, and T3, followed by a COVID-19 assessment of history and timing of COVID-19 testing and diagnosis (T4: ~14 days after T3). Continuous measures were described using mean (SD) values*,* while categorical measures were summarized as n (%) values. Formal comparisons were made on the basis of COVID-19 status. The multivariate model was determined by entering all statistically significant variables (*P*<.05) in univariable associations at once and then removing one variable at a time through backward selection until the optimal model was obtained.

**Results:**

During the fall 2020 semester, 1997 participants consented, enrolled, and met criteria for data analyses. There was a high prevalence of anxiety, as assessed by the State Trait Anxiety Index, with moderate and severe levels in 465 (24%) and 970 (49%) students, respectively. Approximately one-third of students reported having a mental health disorder (n=656, 33%). The average daily steps recorded in this student population was approximately 6500 (mean 6474, SD 3371). Neither reported mental health nor step count were significant based on COVID-19 status (*P*=.52). Our analyses revealed significant associations of COVID-19 positivity with the use of marijuana and alcohol (*P*=.02 and *P*=.046, respectively) and with lower belief in public health measures (*P*=.003). In addition, graduate students were less likely and those with ≥20 roommates were more likely to report a COVID-19 diagnosis (*P*=.009).

**Conclusions:**

Mental health problems were common in this student population. Several factors, including substance use, were associated with the risk of COVID-19. These data highlight important areas for further attention, such as prioritizing innovative strategies that address health and well-being, considering the potential long-term effects of COVID-19 on college students.

**Trial Registration:**

ClinicalTrials.gov NCT04766788; https://clinicaltrials.gov/ct2/show/NCT04766788

**International Registered Report Identifier (IRRID):**

RR2-10.2196/29561

## Introduction

As SARS-CoV-2 spread throughout the United States and worldwide [1], the COVID-19 pandemic disrupted and transformed education overnight [2]. Reacting to the COVID-19 pandemic and subsequent quarantine and isolation measures [3], academic institutions across the nation adapted to virtual learning owing to closures of in-person schooling. The unprecedented changes included significant reduction in access to campus resources (eg, libraries, computing facilities, group study areas, mental health services, and exercise facilities), which upended the education landscape [2,4] and created intense stress across institutions. Several recent studies provide evidence for a high prevalence of mental health problems among college students who experienced virtual education [5-16].

Given the potential profound impact of the COVID-19 pandemic on the health and well-being of college students, our interdisciplinary team leveraged a positive psychology–based mobile health (mHealth) app, Roadmap 2.0, as a resilience-building platform for the student population*.* The Roadmap platform was initially developed to provide support to patients and their family caregivers in health care delivery (eg, information, education, and skills-based training) because of its accessibility and scalability [17-23]. This platform was iteratively enhanced to support the health and well-being of the user and to aggregate their raw step and sleep counts, which were collected through the Fitbit [24].

Herein, this Roadmap platform was leveraged to (1) characterize self-reported outcomes of physical, mental, and social health by COVID-19 status during the fall 2020 semester; (2) assess physical activity through consumer-grade wearable sensors (Fitbit) by COVID-19 status [25]; and (3) evaluate potential risk factors associated with COVID-19 positivity, including student demographics (eg, gender, race, and ethnicity), substance use, and physical, mental, and social health constructs [25]. This work is important because it may inform future mHealth design interventions for this population. Moreover, these data may be important factors to consider when developing future public health responses that include massive disruptions to mitigate spread of communicable diseases, particularly in emerging, young adults. By using the Roadmap platform, we sought to focus our findings on the nexus between mental and social health constructs with physical activity and COVID-19 status.

## Methods

### Study Site

The data coordinating site was a Midwestern academic institution (University of Michigan [U-M]). All study activities were conducted remotely with no in-person contact, and all study materials were mailed to participants’ residences through a US shipping company.

### Study Design, Recruitment, and Informed Consent

The study protocol has been previously published with more details [25]. Briefly, eligibility for study participation included the following: age ≥18 years, being a confirmed undergraduate or graduate U-M student (eg, on campus or at home). being able to provide digital informed consent, being comfortable with reading and speaking English, and having access to necessary resources for participating in an mHealth technology-based intervention (ie, smartphone or tablet device and internet access) while also being willing to use personal equipment or the internet for the study.

**Figure 1:**
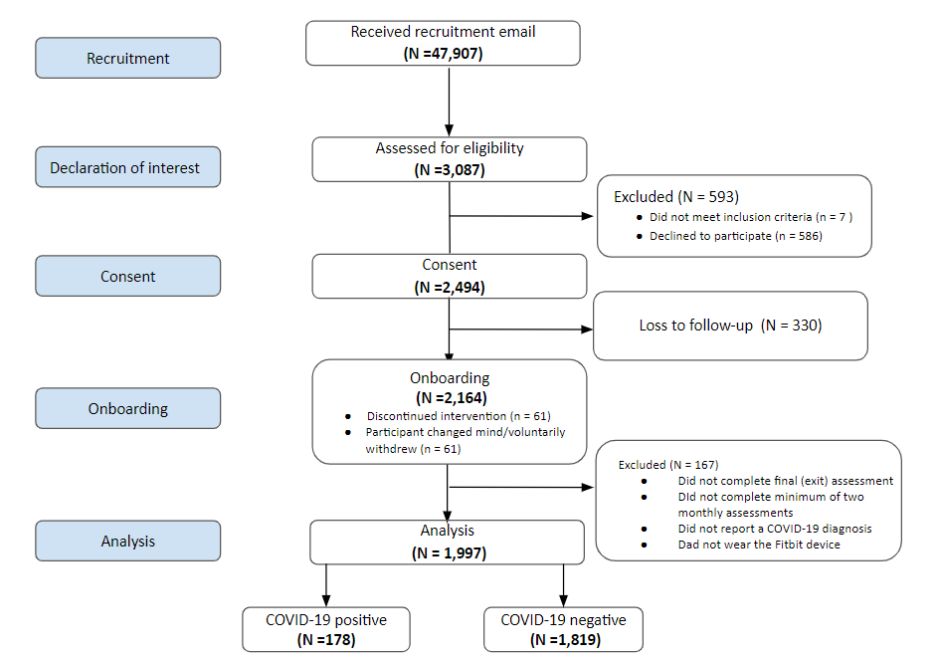
CONSORT (Consolidated Standards of Reporting Trials) flow diagram [26] for participant recruitment and enrollment.

The recruitment period was between September 2020 and December 2020. While paper flyers and postings were distributed throughout the campus buildings, the primary mode of recruitment was by the “Targeted Email and Data Service,” coordinated by the U-M Registrar’s Office, with IRBMED approval (Figure 1).

Interested participants who contacted the study team by telephone or email received additional study information (eg, overview of study procedures, risks, and benefits). Following confirmation of university student status, the research coordinator emailed the informed consent through the SignNow platform, and the participant signed the document electronically [27].

### Study Procedures

The study procedures are outlined in Multimedia Appendix 1.

#### Wearable Device

The Fitbit was mailed to the participants’ homes. They were instructed to use it continuously (at least ~40 hours/week) to measure their physical activity, heart rate, and sleep during the monitoring period. The Fitbit automatically generated accelerometer-based summary data (per proprietary algorithms) that were based on “activity counts” collected over the course of the day. We assessed participant compliance in wearing the Fitbit by identifying when heart rate data were present through the Roadmap platform using the Fitbit API [28]. We measured daily wear time using heart rate data with a minute-level resolution. Compliance was expressed both in hours (0-24 hours) and in percentages (ie, by dividing the number of hours spent wearing the device by 24) [29]. By assessing compliance, we calculated the average daily step count for participants who wore the Fitbit for more than 6 hours between 8 AM and 8 PM. We chose a cut-off of 6 hours because the distribution of average daily step count did not change significantly for higher cut-offs. No compliance cut-off was applied for calculating asleep hours because the daily average changed by only approximately .05 hours between a cut-off of 0 hours and a cut-off of 11 hours between 8 PM and 8 AM.

#### Roadmap and Fitbit Apps

Participants were instructed to download the Roadmap 2.0 (Multimedia Appendix 2) and Fitbit apps on their smartphone device (both free of charge and publicly available on Apple App Store and Google Play).

#### Self-reported Outcomes

All self-reported physical, mental, and social health data were collected using Roadmap 2.0, which used Qualtrics (Qualtrics), a web-based research tool that enables researchers to create study-specific websites for administering study surveys and storing participant data. The data were associated via a unique study participant ID and did not contain any identifying information. Data were stored in the cloud and regularly downloaded and saved on Health Insurance Portability and Accountability Act–compliant and password-protected university servers. Participants were instructed to complete surveys at baseline (preintervention: T0, monthly: T1, T2, T3, and upon study exit [T4] using the Roadmap platform). A list of the survey questionnaires is provided in the Research Protocol [25]. Psychometric properties of these measures are provided in Multimedia Appendix 3. Of note, only the preintervention (T0) mental health and health behaviors data were analyzed in this study. The 9-item Patient Health Questionnaire (PHQ-9; for depression) and the 7-item General Anxiety Disorder (GAD-7; for anxiety) scales were added into the study protocol after the study began, and only a subset of participants answered these items.

### Statistical Analyses

For the descriptive statistics, continuous measures were described using mean (SD) values, while categorical measures were summarized as n (%) values. These data were analyzed using SAS software (SAS Institute). Formal comparisons were made on the basis of COVID-19 status (ie, positive or negative) with Cronbach α levels (statistical significance) set at *P*<.05.

Logistic regression models were fit in two stages. First, univariate associations of student demographics and characteristics, mental health, self-reported substance use and social health measures were assessed by COVID-19 status. Second, the multivariate associations of student demographics and characteristics, mental health, self-reported substance use, and social health measures were assessed by COVID-19 status. The multivariate model was developed by entering all statistically significant variables (*P*<.05) in univariable associations at once and then removing one variable at a time by backward selection until the optimal model was obtained (ie, the deviance of the model was minimized).

Next, to test the performance of the multivariate regression model, several receiver operating characteristic (ROC) curves were plotted for candidate models: Model 1 included only demographic variables; Model 2 included demographic and mental health measures; Model 3 included demographic, mental health measures, and self-reported substance use; and Model 4 (Full Model) included all the variables in Models 1 through 3 plus all other significant characteristics and social health variables from univariate associations; as well as Model 5 (Final Model), which was selected through backward stepwise regression from Model 4. Model 5 provided the minimal Akaike Information Criterion. The area under the ROC curve (AUC) represented the prediction accuracy of the current model. When we constructed our models, we observed an increase in accuracy as more variables were added from Model 1 to Model 4. Importantly, even though Model 5 included fewer variables than the Model 4, we did not observe a significant loss in prediction accuracy. Thus, Model 5 was selected as the final multivariate model owing to its simplicity. The univariate and multivariate logistic regression were analyzed using R (version 4.1.1). Figures and graphs were generated with GraphPad Prism (version 9.1.0 for Windows, GraphPad).

### Ethics Approval

 Ethical approval for this study was obtained by the U-M Medical School Institutional Review Board (IRBMED), and the study was registered on ClinicalTrials.gov (NCT04766788).

## Results

### Participant Demographics by COVID-19 Status

The majority of students consented and enrolled in the study during the months of October and November 2020 (Multimedia Appendix 4), which coincided with the peak number of confirmed cases of COVID-19 at the local, state, and national levels (Multimedia Appendix 5). As shown in Table 1, the student population (total N=1997) consisted of undergraduate (n=1312, 66%) and graduate students (n=670, 34%). The majority of the respondents were female (n=1367, 68%) and White (n=1150, 58%), followed by Asian (n=597, 30%), 2 or more races (n=107, 5%), and Black (n=85, 4%). In total, 10% of participants reported their ethnicity as Hispanic or Latinx, and 8% were international students. Approximately one-fourth of the participants were first-generation college students.

In this population, 178 (8.9%) students reported a positive COVID-19 diagnosis (COVID-19 positivity), which occurred either before or during the study period (ie, reported at the baseline, monthly, or the exit survey). These individuals were more likely to be non-Asian, non–multi-racial, domestic undergraduate students, living with ≥20 housemates, or owning iPhone devices.

**Figure 2:**
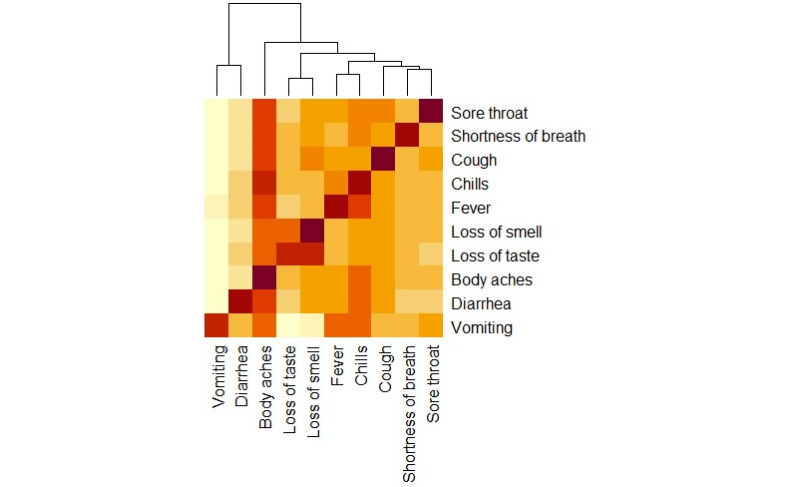
COVID-19 symptoms. The most common clusters of associated dyadic symptoms were chills and body aches (cluster 1, n=59), and loss of taste (ageusia) and anosmia (cluster 2, n=49). The most common triad of symptoms was fever, chills, and body aches (cluster 3, n=40). Body chills occurred most frequently, which was concurrently most frequent. All respiratory symptoms (eg, cough, shortness of breath, and sore throat) were associated with one another.

The most common COVID-19 symptoms reported by students included body aches (n=93, 51%), loss of smell (anosmia; n=68, 37%), chills (n=67, 36.8%), and cough (n=64, 35%). Additionally, the most common clusters of associated dyadic symptoms were chills and body aches (Cluster 1: n=59) and loss of taste (ageusia) and anosmia (Cluster 2: n=49). The most common triad of symptoms were fever, chills, and body aches (Cluster 3: n=40). Not surprisingly, all respiratory symptoms (eg, cough, shortness of breath, and sore throat) were associated with each other (Figure 2). However, 53 participants (30% of the 178 COVID-19–positive participants) reported that they were asymptomatic.

**Table 1:** Participant demographics and characteristics by COVID-19 status.

Demographics		Population, n (%)	COVID-19–negative students, n (%)	COVID-19–positive students, n (%)	*P* value^a^	
**School year**						<.001
	Freshman	231 (11.6)	209 (90.5)	22 (9.5)		
	Sophomore	355 (17.8)	308 (86.8)	47 (13.2)		
	Junior	338 (16.9)	299 (88.5)	39 (11.5)		
	Senior	388 (19.9)	357 (92.0)	31 (8.0)		
	First year graduate	238 (11.9)	218 (91.6)	20 (8.4)		
	Second year or greater graduate	432 (21.6)	413 (95.6)	19 (4.4)		
**Gender**						.92
	Female	1367 (68.5)	1244 (91.0)	123 (9.0)		
	Male	613 (30.7)	559 (91.2)	51 (8.8)		
	Other	16 (0.8)	15 (93.7)	1 (6.3)		
**Race**						<.001
	White	1150 (58.1)	1016 (88.4)	124 (11.6)		
	Black or African American	85 (4.3)	80 (94.1)	5 (5.9)		
	American Indian/Alaska Native	4 (0.2)	3 (75.0)	1 (25.0)		
	Asian	597 (30.2)	570 (95.5)	27 (4.5)		
	Multiracial	107 (5.4)	102 (95.3)	5 (4.7)		
	Other	37 (1.9)	32 (86.5)	5 (13.5)		
**Ethnicity**						.13
	Hispanic or Latino	193 (9.7)	170 (88.1)	23 (11.9)		
	Non-Hispanic or Latino	1800 (90.3)	1645 (91.4)	155 (8.6)		
**Domestic or international**						.01
	Domestic	1843 (92.4)	1670 (90.6)	173 (9.4)		
	International	151 (7.6)	146 (96.7)	5 (3.3)		
**First or continuing generation**						.60
	First generation	503 (25.3)	461 (91.7)	42 (8.3)		
	Continuing generation	1489 (74.7)	1353 (90.9)	136 (9.1)		

^a^*P* values are representative of a chi-square test performed for the entire study population.

### Self-reported Mental and Social Health by COVID-19 Status

A high prevalence of anxiety, as assessed by the State Trait Anxiety Index (STAI), was reported at moderate (n=465, 24%) and severe (n=970, 49%) levels in this student population (Table 2). These findings were consistent with those of self-reported anxiety (n=570, 28%), depression (n=373, 19%), or indication of any mental health disorder (n=656, 33%) when asked the question, “do you have any of the following health conditions?” However, there were no differences in these parameters in accordance with COVID-19 status. Similarly, there were no differences in levels of coping, compassion, or flourishing between the groups in accordance with COVID-19 status. Not surprisingly, this population reported high levels (mean 6.14, SD 0.88; maximum 7.0) of desire for academic success. Interestingly, lower levels of loneliness and higher social fitness were associated with COVID-19 positivity (Table 2).

Given the high prevalence of anxiety in our population, we were interested in examining their coping levels. Table 3 details the mean scores on the Brief Coping Orientation to Problems Experienced inventory based on problem-focused, emotion-focused, and avoidant coping subscales [30]. As a population, students had the highest mean scores for acceptance, followed by self-distraction, and the lowest mean scores for denial and substance use. Low levels of planning and higher use of humor and substance use were associated with COVID-19 positivity (Table 3). Pearson correlation analysis revealed a significant negative association between anxiety and compassion (*r*=–0.22) as well as anxiety and flourishing (*r*=–0.71). There was also a significant positive correlation between compassion and flourishing (*r*=0.22). There was no relationship between compassion and adherence to public health COVID-19 measures (*r*=–0.001; Multimedia Appendix 6).

**Table 2:** Self-reported mental health outcomes by COVID-19 status.

Mental health outcome	Population, mean (SD)	COVID-19–negative students, mean (SD)	COVID-19–positive students, mean (SD)	*P* value
State Trait Anxiety Index trait	44.49 (10.61)	44.55 (10.60)	43.86 (10.78)	.41
Compassion	3.46 (0.91)	3.46 (0.92)	3.46 (0.87)	.95
Flourishing	7.35 (1.47)	7.34 (1.46)	7.51 (1.54)	.14
Loneliness	1.94 (0.58)	1.95 (0.58)	1.83 (0.61)	<.001
Social fit	5.03 (1.14)	5.00 (1.13)	5.29 (1.13)	<.001
Academic success	6.14 (0.88)	6.13 (0.87)	6.21 (0.95)	.29

**Table 3:** Outcomes on the Brief Coping Orientation to Problems Experienced inventory by COVID-19 status.

Coping mechanisms		Population, mean (SD)	COVID-19–negative students, mean (SD)	COVID-19–positive students, mean (SD)	*P* value
**Problem-focused coping**		2.46 (0.59)	2.47 (0.59)	2.40 (0.57)	.14
	Active coping	2.45 (0.76)	2.45 (0.76)	2.37 (0.72)	.16
	Instrumental support	2.39 (0.86)	2.40 (0.86)	2.34 (0.83)	.38
	Positive reframing	2.48 (0.83)	2.47 (0.83)	2.54 (0.82)	.30
	Planning	2.53 (0.80)	2.55 (0.80)	2.36 (0.80)	<.001
**Emotion-focused coping**		2.34 (0.42)	2.34 (0.42)	2.34 (0.40)	.92
	Emotional support	2.64 (0.88)	2.64 (0.89)	2.60 (0.86)	.59
	Venting	2.14 (0.72)	2.14 (0.73)	2.13 (0.69)	.79
	Humor	2.29 (0.92)	2.28 (0.92)	2.44 (0.86)	.02
	Acceptance	3.22 (0.68)	3.23 (0.67)	3.17 (0.67)	.21
	Self-blame	2.04 (0.81)	2.04 (0.81)	2.07 (0.85)	.75
	Religion	1.67 (0.89)	1.67 (0.90)	1.63 (0.83)	.51
**Avoidant coping**		1.77 (0.38)	1.76 (0.38)	1.84 (0.41)	.008
	Self-distraction	2.96 (0.72)	2.95 (0.72)	2.99 (0.70)	.49
	Denial	1.20 (0.44)	1.20 (0.43)	1.26 (0.48)	.10
	Substance use	1.40 (0.69)	1.38 (0.66)	1.62 (0.84)	<.001
	Behavioral disengagement	1.53 (0.65)	1.53 (0.64)	1.49 (0.68)	.47

### Self-reported Substance Use by COVID-19 Status

Among all students, cigarette smoking was low (n=23, 1.2%), while the numbers of students who reported any marijuana use, vaping, and alcohol use were 847 (42.6%), 431 (21.6%), and 1600 (80.4%), respectively, which were all associated with COVID-19 positivity (Table 4). Moreover, students who reported a mental health problem were significantly more likely to use marijuana (odds ratio [OR] 1.76, 95% CI 1.46-2.13), consume alcohol (OR 2.22, 95% CI 1.70-2.90), engage in vaping (OR 1.64, 95% CI 1.32-2.04), or smoke cigarettes (OR 4.76, 95% CI 1.95-11.63; Multimedia Appendix 7).

**Table 4:** Health behaviors including substance use and exercise by COVID-19 status.

Health behaviors			Population, n (%)		COVID-19–negative students, n (%)		COVID-19–positive students, n (%)		*P* value	
**Marijuana**										<.001
	Yes	847 (42.6)		737 (87.0)		110 (13.0)				
	No	1143 (57.4)		1076 (94.1)		67 (5.9)				
**Smoking**										.49
	Yes	23 (1.2)		20 (87.0)		3 (13.0)				
	No	1973 (98.8)		1798 (91.1)		175 (8.9)				
**Vaping**										<.001
	Yes	431 (21.6)		359 (83.3)		72 (16.7)				
	No	1563 (78.4)		1458 (93.3)		105 (6.7)				
**Alcohol consumption**										<.001
	Yes	1600 (80.4)		1435 (89.7)		165 (10.3)				
	No	391 (19.6)		379 (20.9)		12 (3.1)				
**Exercise**										.42
	Yes	1917 (96.0)		1745 (91.0)		172 (9.0)				
	No	79 (4.0)		74 (93.7)		5 (6.3)				

### Using Fitbit Data to Assess Physical Health in College Students by COVID-19 Status

**Figure 3:**
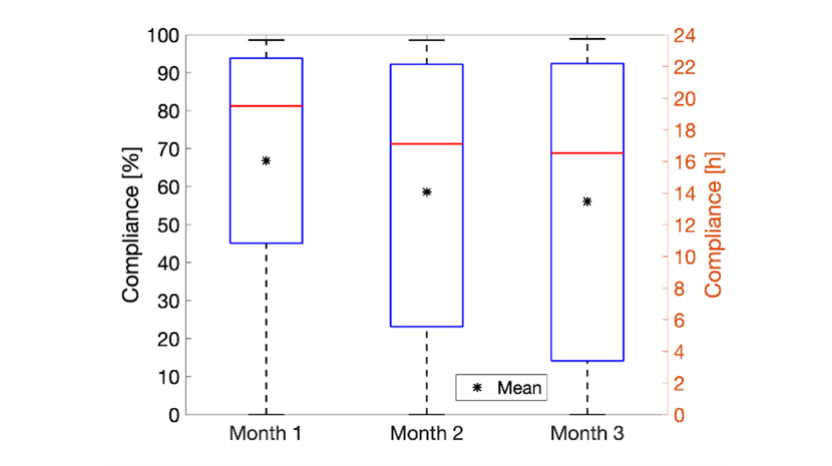
Fitbit compliance over time. Each boxplot represents the daily compliance averaged chronologically for each 30-day span of the 90-day study period for all participants. Error bars indicate the minimum and maximum values.

In addition to completing longitudinal survey measures, students also provided continuous physiological data by wearing the Fitbit device throughout the study period. The average wear time of the device was 14.5 hours (in a 24-hour day), 7.4 hours during daytime (between 8 AM and 8 PM), and 7.1 hours during nighttime (between 8 PM and 8 AM). As shown in Figure 3, we observed a modest decline in compliance over the 90 days of the study, from an average of 16.1 hours for the first 30 days, to 13.5 hours for the last 30 days. Students who reported COVID-19 positivity had significantly lower average daily compliance (24 hours) than those who did not (*P*=.04). Multimedia Appendix 8 shows the distribution of average daily compliance by COVID-19 status. The average number of daily steps in this student population was approximately 6500 (mean 6474, SD 3371; Multimedia Appendix 9). There were no significant differences in average daily step counts in accordance with COVID-19 status (*P*=.52).

### Multivariate Risk Factors for COVID-19 Positivity

In the final multivariate model of student demographics and characteristics, including physical, mental, and social health variables, individuals who reported marijuana or alcohol use and lived with a greater number of housemates (≥20) were at increased risk of COVID-19 positivity. However, being a graduate student and being an individual who aligned with public health measures were associated with COVID-19 negativity (Table 5). Graduate students’ protection from COVID-19 may have resulted from their less dense living environments compared to those of undergraduate students (*P*<.001). Model 5 with an AUC of 85% is available in Multimedia Appendix 10. All models that were significant at the univariate level and included in the multivariate model are shown in Multimedia Appendix 11.

**Table 5:** The final multivariate model.

Predictor				Estimate (SE)		*P* value
**Demographics**						
	Intercept		–1.9116 (1.0022)		.06	
	**Race**					
		Black or African American	0.0170 (0.5414)		.98	
		American Indian/Alaska Native	1.5975 (1.2206)		.19	
		Asian	–0.4895 (0.2723)		.07	
		Multiracial	0.9844 (0.5664)		.08	
		Other	–1.1114 (0.6125)		.07	
	**Grade**					
		Sophomore	0.0873 (0.3488)		.80	
		Junior	–0.0834 (0.3512)		.81	
		Senior	–0.5921 (0.3686)		.11	
		Graduate student (first year)	–0.9850 (0.4520)		*.03* ^a^	
		Graduate student (second year)	–1.1648 (0.4160)		*.01*	
		Other	–14.1032 (596.5290)		.98	
**Mental**						
	Coping: planning (planning)		–0.2215 (0.1269)		.08	
**Substance use**						
	Marijuana (binary usage)		0.5523 (0.2387)		*.02*	
	Alcohol (binary usage)		0.7483 (0.3756)		*.046*	
	Vaping (binary usage)		0.3807 (0.2342)		.10	
**Other**						
	Student social fit (numeric)		0.1774 (0.0988)		.07	
	Public health beliefs (numeric)		–0.2219 (0.0752)		*.003*	
	Loneliness (numeric)		–0.3217 (0.1902)		.09	
	**Belief in COVID-19 likeliness**					
		Somewhat agree	0.1460 (0.5055)		.77	
		Neither agree nor disagree	0.3415 (0.4947)		.49	
		Somewhat disagree	0.5355 (0.4869)		.27	
		Strongly disagree	0.7695 (0.5485)		.16	
		Already had COVID-19	6.7463 (0.8943)		*<.001*	
	**Number of housemates**					
		1-3	–0.1159 (0.2905)		.69	
		3-10	–0.4007 (0.3362)		.23	
		10-20	0.6635 (0.5937)		.26	
		>20	1.3433 (0.5160)		*.009*	

^a^Italicized *P* values indicate significance at *P*<.05.

## Discussion

### Principal Results

A major finding in this study indicates concerns of adverse mental health symptoms reported by college students, confirming data from other recent studies [31-34]. When looking at the STAI trait, 73% of our study population had moderate or severe anxiety. Our study also used the GAD-7 and PHQ-9 assessments of anxiety and depression, respectively, in a subset of our students. The data not shown indicate that approximately 52% of participants (among those who completed the GAD-7, n=1366) reported having anxiety and 65% of participants (among those who completed the PHQ-9, n=1365) reported having depressive symptoms. These data further highlight the high prevalence of mental health problems in current college students [35]. Indeed, the upsurge in mental health problems among college students has escalated to alarming levels nationwide [32,34,36], which was likely amplified by the effects of the global pandemic [2,15].

Our study did not find a difference in mental health data reported by COVID-19 status, whereas substance use was significantly associated with COVID-19 positivity. It is possible that in some students who reported mental health problems, their coping strategies may have included substance use behaviors (eg, marijuana and alcohol consumption), which tend to be social activities occurring in groups (ie, more than one individual). Indirectly, this may have accounted for increased COVID-19 risk owing to less vigilant safety practices. Alternatively, among other students who reported mental health problems, those problems may be associated with (or be due to) isolation, thereby decreasing their COVID-19 risk. It is possible this competing process canceled out any significant total effect of mental health problems by COVID-19 status.

### Comparison With Prior Work

This study leveraged mHealth technology to characterize the demographics and physical, mental, and social health of college students during a global pandemic. During a unique period in history where all in-person research activities were halted, the mHealth platform facilitated this type of data collection. The findings herein were self-reported by students prior to the availability of COVID-19 vaccines nationwide. Approximately 9% of students who participated in this study reported COVID-19 positivity. Across the nation, there were over 30 million cumulative reported positive COVID-19 cases by April 01, 2021 [37], which was approximately 9.2% of the US population. In the state of Michigan, where this study was conducted, ~750,000 COVID-19 cases were reported by this time (~7.5% of the population). In addition, cases in Michigan have been most prevalent in the 20-29–year age group [38], which may be owing to students living in close proximity during the pandemic.

In our sample of college students, the most common symptoms were body aches, anosmia, chills, and cough. Interestingly, in a large meta-analysis of 9 countries and 24,410 adults, the most commonly reported symptoms were fever (78%) and cough (58%) [39]. However, many of the studies contributing to this meta-analysis were focused on patients requiring hospitalization, which suggests that these symptoms may have been more common in infections with severe clinical phenotypes. Another recent study used an mHealth app that reported symptomology and COVID-19 test results in ~3.2 million users. Within the symptomatic population, 60.4% reported a cough, while only 42.7% reported a fever [40]. Those data were consistent with our findings in that 51.2% of our symptomatic population reported cough and 45.6% reported fever.

We found that second-year graduate students, Asians and multi-racial students, and international students were significantly less likely to report COVID-19 positivity. There was also an association between an increased number of roommates and an increased risk of COVID-19 positivity. Graduate students in our sample lived with significantly fewer people, presumably decreasing their risk of COVID-19. Marijuana and alcohol consumption were significant risk factors for COVID-19. Additionally, students who agreed or believed in public health measures were less likely to report COVID-19 positivity.

Not surprisingly, we observed a relatively modest decrease in Fitbit compliance over the study duration. This was likely due to decreased engagement with both the Fitbit device and the study over time. Despite the ease of use of consumer-grade wearable sensors, “wearables abandonment” is a well-documented issue [41]. Nonetheless, we observed a large proportion of highly compliant students (ie, daily wear time >14 hours) and a smaller proportion with lower levels of compliance (<2 hours). Students who reported COVID-19 positivity showed a bimodal-like distribution in their Fitbit compliance compared to COVID-19–negative students. The NetHealth study recorded overall higher levels of compliance for a longer period on using a similar Fitbit device in a college student population [29]. However, the NetHealth study was conducted from 2015 to 2017 (ie, prior to the one of the COVID-19 pandemic), which may help explain the different behaviors. Additionally, the differences may be attributed to the compensation model of the studies. The study herein did not incentivize regular data reporting outside of providing the Fitbit device, whereas the NetHealth study did (ie, it provided monetary compensation for regular Fitbit use and data reporting).

The average daily step count observed in this study population was 6474 (SD 3371), which was a relatively low level of physical activity [42]. The NetHealth study conducted with 692 college students reported an average prepandemic daily step count of 11258 (SD 5874) [43]. This large difference in physical activity was likely due to pandemic procedures and norms (eg, isolation, quarantine, and public health guidelines). Specifically, during that time the periodic walks to and from classes (on campus) were possibly limited by the virtual learning environment and strict isolation and quarantine guidelines mandated by the university when the number of COVID-19 cases had peaked Multimedia Appendix 5. Of note, data analyses are forthcoming in examining the impact of Roadmap’s resilience-based activities on physical, mental, and social health outcomes over time (pre- and post-), given the study’s longitudinal design. Moreover, we currently have a “Re-contact Student Study” (postvaccination era) in the same study population of students who participated in the initial 2020-2021 Student Study, which will allow us to compare data from the pre- and postvaccination eras in future analyses.

### Limitations

We interpret the findings herein within the context of several limitations. Owing to the single-institution design, our findings are not generalizable outside of our student cohort. In the fall 2020 semester, the undergraduate U-M student population was represented by the following racial and ethnic categories: White (n=17,307, 55.2%), Asian (n=5111, 16.3%); international (n=2301, 7.4%); Hispanic or Latinx (n=2187, 7%); not indicated (n=1615, 5.2%); 2 or more (n=1508, 4.8%); Black or African American (n=1249, 4%); American Indian/Alaska Native (n=36, 0.11%); and Native Hawaiian/Other Pacific Islander (n=14, 0.04%). While our cohort recruited similar proportions of racial or ethnic categories that are reflective of the U-M student demographics, there was a greater percentage of Asian students. In addition, females were much more likely to participate in our study than males (68% vs 31%), despite a roughly equal percentage of gender types attending the U-M (50.3% female, 49.7% male).

Second, data attrition resulting from wearable abandonment or digital fatigue may have underestimated the student population’s physical activity based on their daily step count. Future work involving emerging adults should consider types of compensation models to incentivize engagement [29] as well as study designs that are adaptive rather than a one-size-fits-all approach [44]. Lastly, COVID-19 diagnosis and symptoms were based on self-report. Our study was limited by resources that did not allow for routine surveillance testing during the study period or access to student medical records to confirm a COVID-19 diagnosis and symptoms reporting.

Despite these limitations, our study had robust recruitment during a period wherein face-to-face research activities were largely halted, indicating the feasibility and merit of conducting a longitudinal study of this nature during a global pandemic. Mobile health technology enabled the team to conduct a virtual and contactless study from recruitment, informed consent, enrollment, onboarding, and multiparameter data collection, including self-report measures and physiological data. The study design aligned with student preferences regarding their ease of use of technology, such as the use of Fitbits and smartphones.

### Conclusions

In summary, our results provide initial data supporting the use of an mHealth platform during a global pandemic, while in-person activities were significantly altered. The most significant factors associated with the risk of COVID-19 positivity in this population included student demographics (eg, graduate student and number of roommates), behavioral factors (eg, marijuana use and alcohol consumption), and beliefs in public health measures. Soberingly, a substantial proportion of this student population was facing a mental health problem and substance use was common. Over the course of the COVID-19 pandemic, students’ educational opportunities have been abruptly disrupted, which may have long-term, unintended consequences. Thus, attention to the current student mental health crisis is imperative with an urgent need to develop novel and timely interventions that address student health and well-being [2].

## Appendix

Multimedia Appendix 1 Study Procedures. Recruitment occurred in college students that were 18 years or older through a targeted email and posted flyers. Interested participants reached out through email and study coordinators performed an informed consent process. Participants were then onboarded via HIPAA (Health Insurance Portability and Accountability Act)-approved teleconference (e.g., Zoom) or recorded video. Onboarding included downloading the Roadmap 2.0 and Fitbit apps as well as completing the baseline survey. Participants were instructed to continue Fitbit syncing and monthly surveys for the next 3 months. Two weeks after the final monthly survey, students took one more closing survey and Fitbit data were collected to the end of the academic year.

Multimedia Appendix 2 Main components of the roadmap app.

Multimedia Appendix 3 Psychometric properties of self-report measures.

Multimedia Appendix 4 Student enrollment over time.

Multimedia Appendix 5 COVID-19 Cases at the local (University of Michigan and Washtenaw County), state (Michigan), and national (U.S.) levels. University: UM Covid-19 data. Campus Maize and Blueprint. 2021. https://campusblueprint.umich.edu/dashboard. County and State: Michigan.gov. 2021. Coronavirus. https://www.michigan.gov/coronavirus. United States: Center for Disease Control. 2021. COVID data tracker. https://covid.cdc.gov/covid-data-tracker/#trends_dailytrendscases.

Multimedia Appendix 6 Correlations of flourishing, compassion, State Trait Anxiety Index (STAI) trait, and public health beliefs.

Multimedia Appendix 7 Odds Ratios of substance use where no report of a mental health condition is the reference group compared to those who reported any mental health condition.

Multimedia Appendix 8 Distribution of compliance by COVID-Status – Distribution of the average daily compliance per participant by COVID-status. The mean and standard deviation of each distribution are given.

Multimedia Appendix 9 Distribution of the average daily step count over the 90-day study period. The mean and standard deviation of each distribution are given.

Multimedia Appendix 10 Model selection. Model (1) included only demographic variables; Model (2) included demographic and mental measures; Model (3) included demographic, mental measures, and self-reported substance use; and (4) the Full Model included all the variables in models (1) through (3) plus all other significant characteristics and social health variables from univariate associations as well as (5) the Final Model, which was selected by backward stepwise regression from the Full Model (4). The Final Model provided the minimal Akaike information criterion (AIC). The Area Under the receiver operating characteristic curve (AUC) represented the prediction accuracy of the current model. When we constructed our models, we observed an increase in accuracy as more variables were added from Model (1) to Model (4). Importantly, even though the Final Model (5) included fewer variables than the Full Model (4), we did not observe a significant loss in prediction accuracy. Thus, the Final Model (5) was selected as the final multivariate model due to its simplicity.

Multimedia Appendix 11 Univariate significant models.

## Abbreviations

AUC: area under the receiver operating characteristic curve
GAD-7: General Anxiety Disorder, 7 items
IRBMED: University of Michigan Medical School’s Institutional Review Board
mHealth: mobile health
NIH/NHBLI: National Institutes of Health’s National Heart, Lung, and Blood Institute
PHQ-9: Patient Health Questionnaire, 9 items
ROC: receiver operating characteristic
STAI: State Trait Anxiety Index
U-M: University of Michigan
