# Supplementary figures and images for: Risk Factors for COVID-19 in College Students Identified by Physical, Mental, and Social Health Reported During the Fall 2020 Semester: Observational Study Using the Roadmap App and Fitbit Wearable Sensors
Source: JMIR Ment Health. 2022 Feb 10;9(2):e34645. doi: 10.2196/34645 (PMC8834863; doi:10.2196/34645)

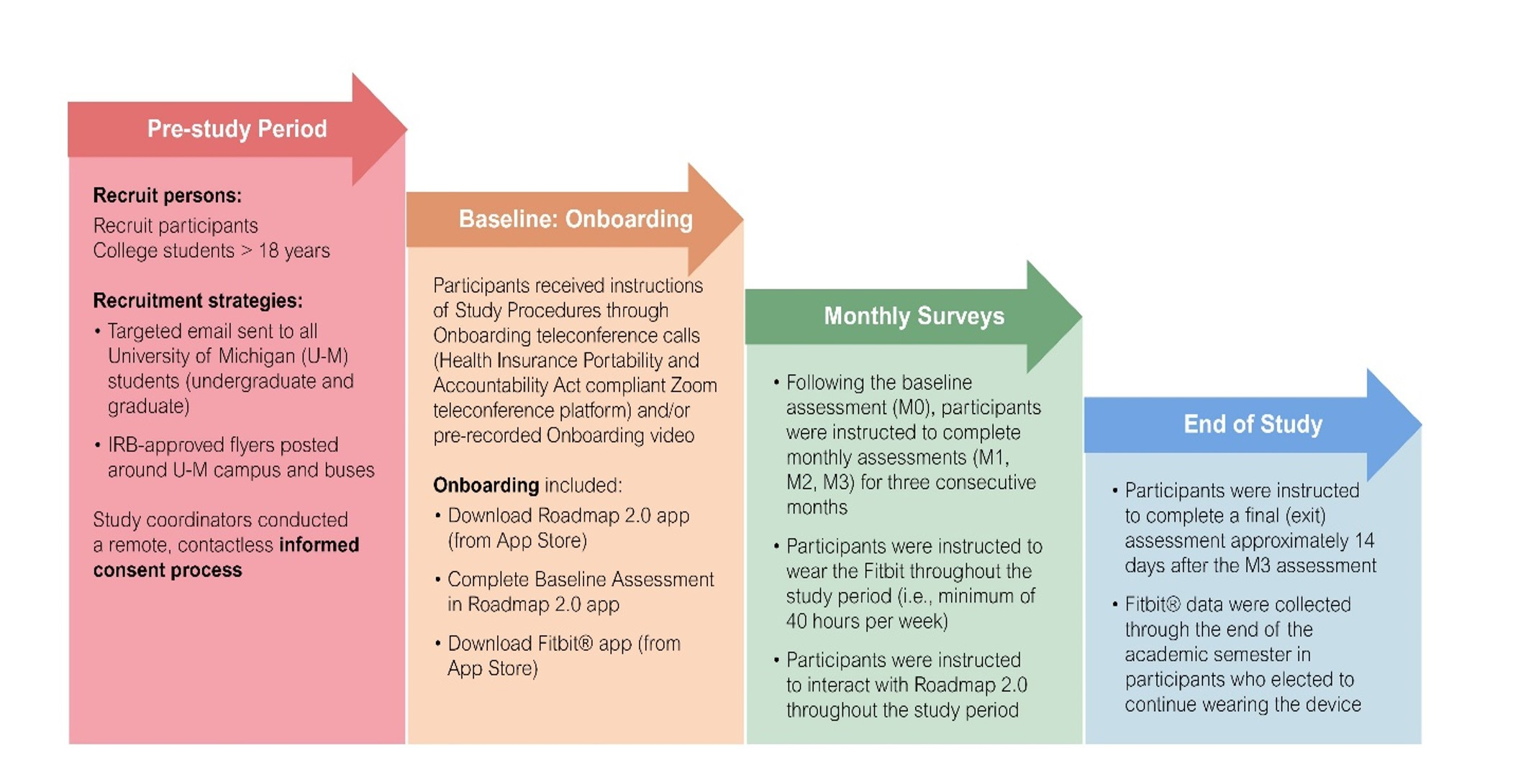

Supplement: Multimedia Appendix 1 [file mental_v9i2e34645_app1.png]

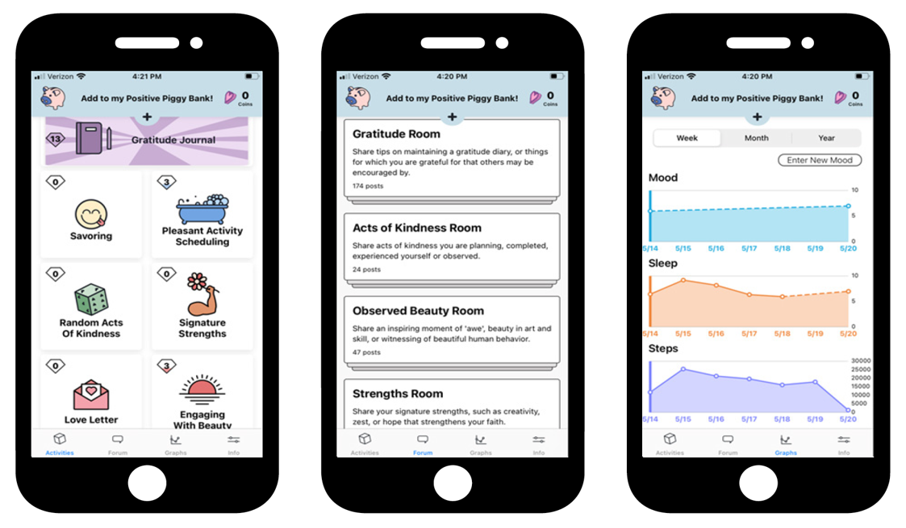

Supplement: Multimedia Appendix 2 [file mental_v9i2e34645_app2.png]

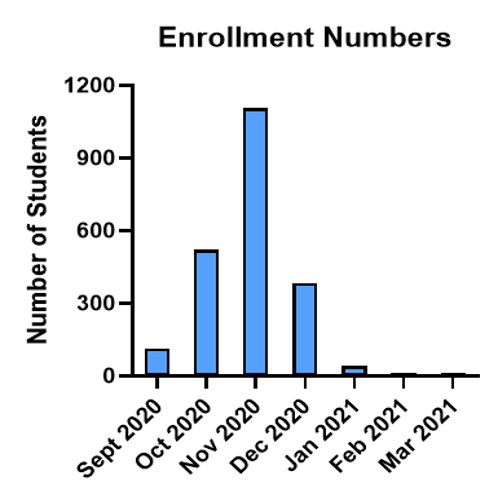

Supplement: Multimedia Appendix 4 [file mental_v9i2e34645_app4.png]

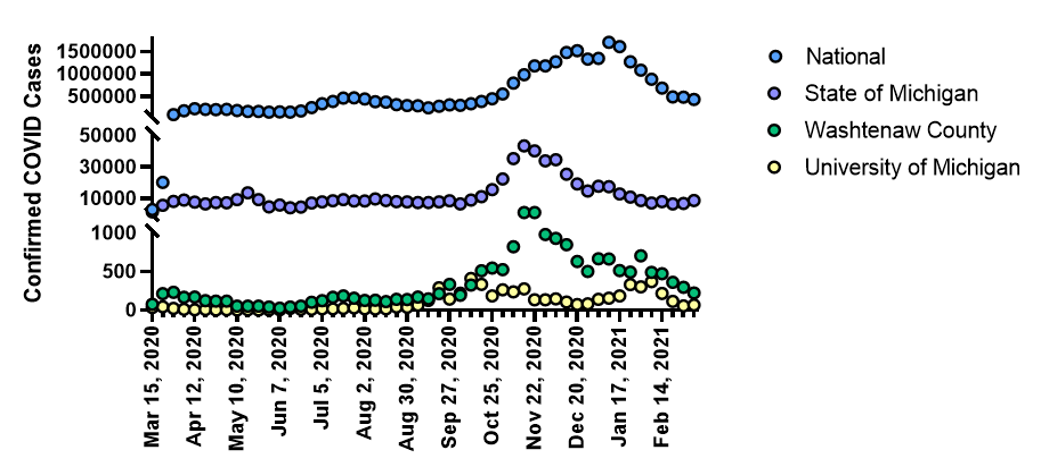

Supplement: Multimedia Appendix 5 [file mental_v9i2e34645_app5.png]

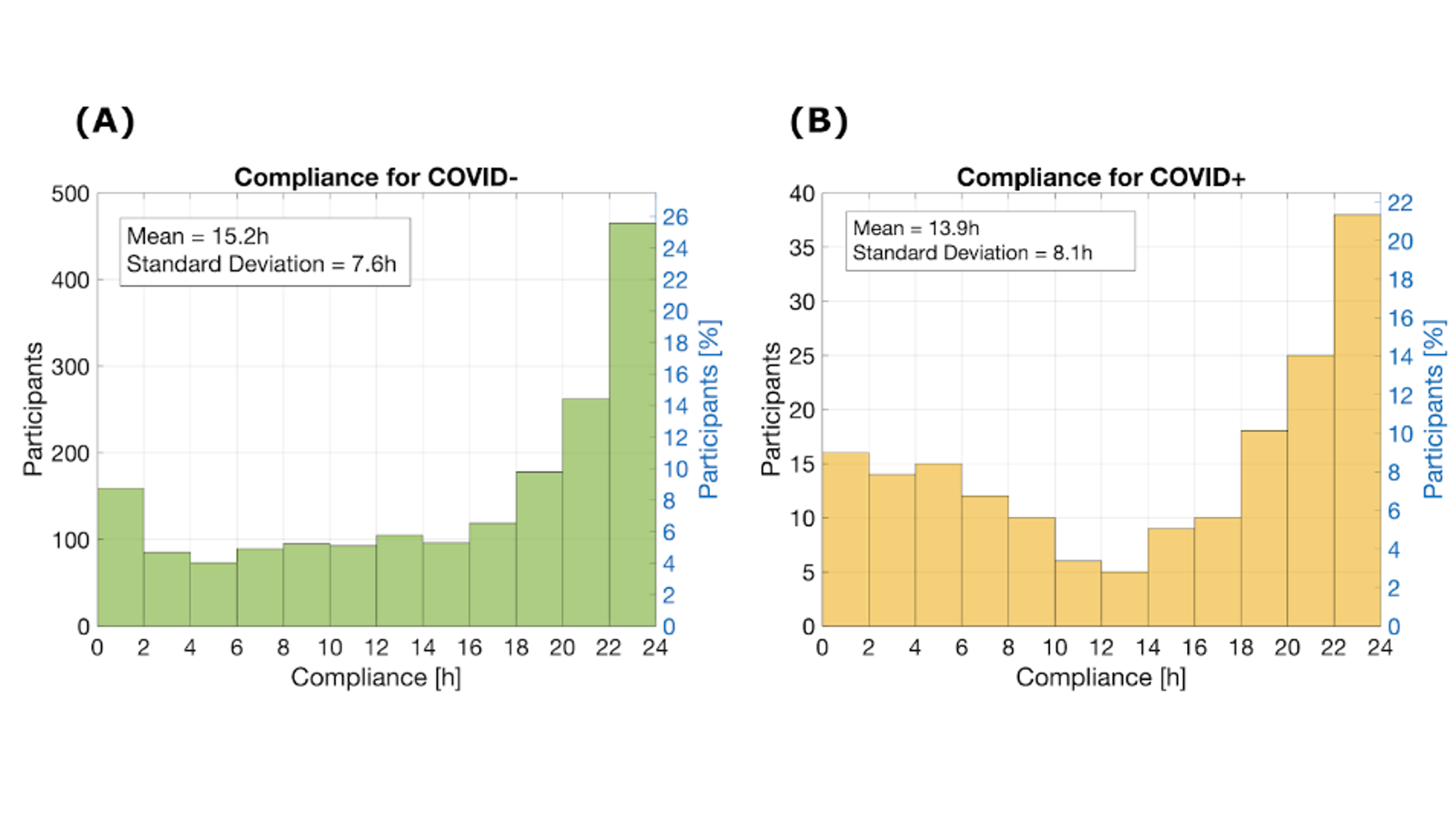

Supplement: Multimedia Appendix 8 [file mental_v9i2e34645_app8.png]

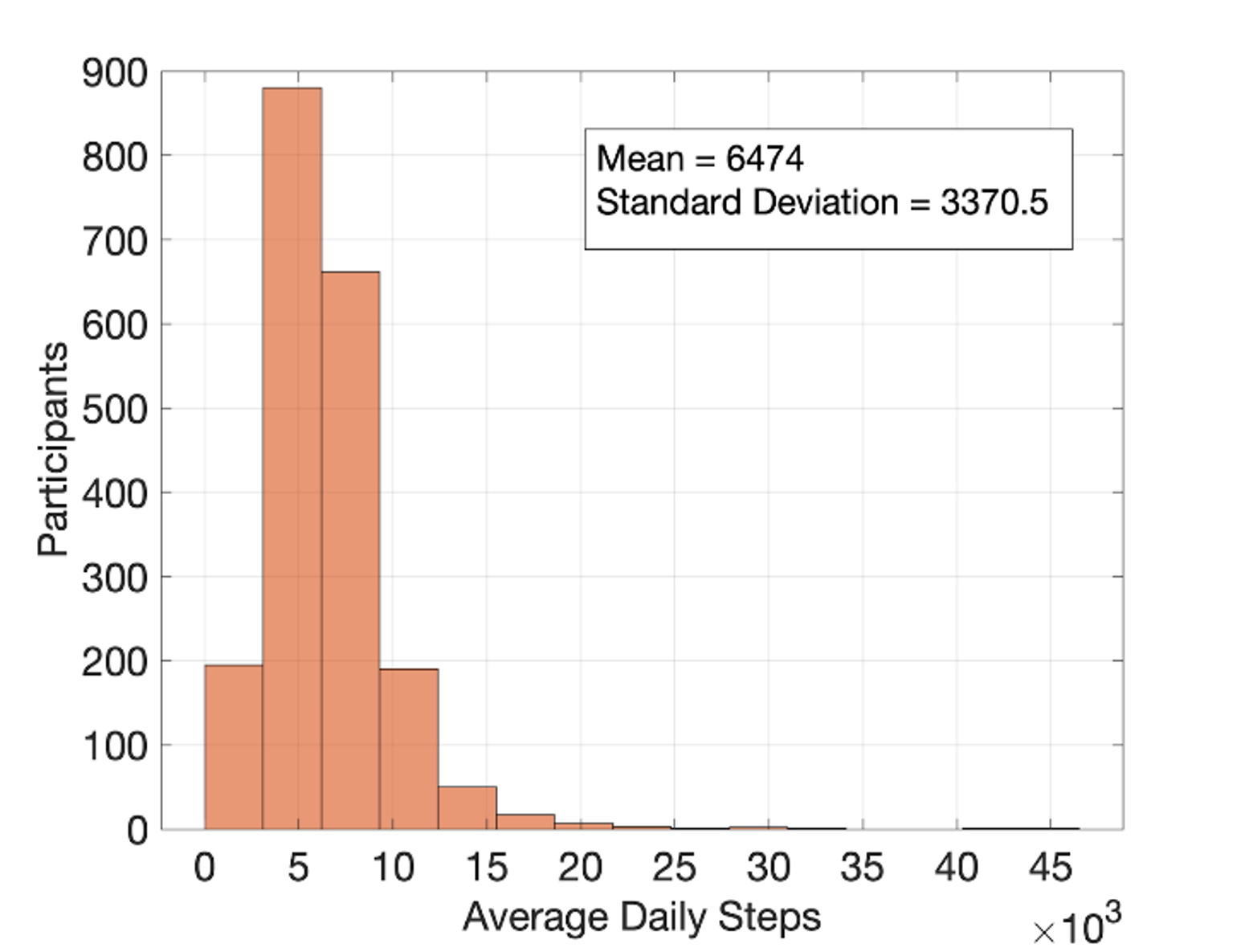

Supplement: Multimedia Appendix 9 [file mental_v9i2e34645_app9.png]

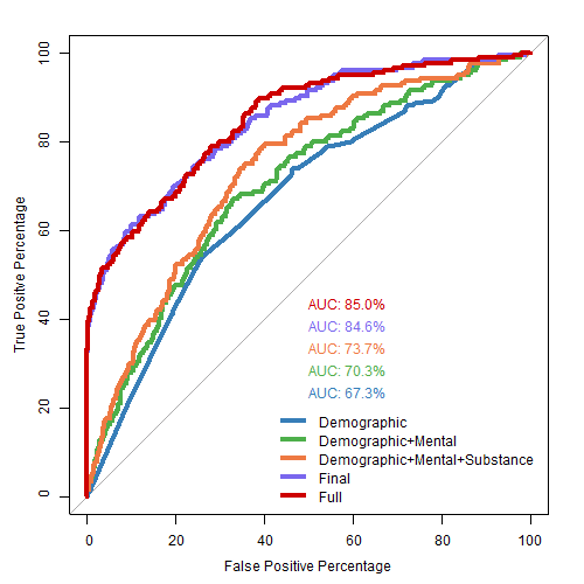

Supplement: Multimedia Appendix 10 [file mental_v9i2e34645_app10.png]
