# Supplementary material for: Risk Factors for COVID-19 in College Students Identified by Physical, Mental, and Social Health Reported During the Fall 2020 Semester: Observational Study Using the Roadmap App and Fitbit Wearable Sensors
Source: JMIR Ment Health. 2022 Feb 10;9(2):e34645. doi: 10.2196/34645 (PMC8834863; doi:10.2196/34645)
Supplement: Multimedia Appendix 3 [file mental_v9i2e34645_app3.doc]

**Multimedia Appendix 3. Psychometric properties of self-report measures.**

| **Questionnaire** | **Cronbach Alpha** | **Description and Validity** |
| --- | --- | --- |
| STAI Trait | 0.92 | A 20-item measure of state anxiety that may be used in clinical settings [1]. Internal consistency for the scale ~0.86 – 0.95 [1-2]. Increased score correlates with increased anxiety. |
| Compassion | 0.83 | A 12-item measure assessing compassion and mental energy, emotional energy [3] Herein we used the main subset which is 4-items. Higher score indicates higher compassion. |
| Flourishing | 0.88 | A 10-item measure with 2-items each in the following categories 1) happiness and life satisfaction 2) mental and physical health 3) meaning and purpose 4) character and virtue 5) close social relationships [4] . Higher score indicates greater flourishing. |
| Loneliness | 0.78 | A 3-item measure assessing isolation, feeling left out, or lacking companionship. A higher score is associated with increased loneliness. |
| Social Fit | 0.87 | A 6-item measure assessing fitness and belonging at the University [5]. A higher score indicates increased perception of social fit. |
| Academic Importance | N/A | A single-item measure assessing, *“How important is academic success to you?”* An increased score indicates increased academic importance. |
| Brief Cope | N/A | A 28-item measure assessing the ability to cope [6]; the current study’s analysis followed the sub-scale analyses provided in Poulus et al 2020 [7]. |
| Belief in Public Health | 0.89 | A 4-item measure assessing the importance of public health measures (adapted from (Kachanoff et al., 2020.) [8]. An increased score indicates increased belief in public health measures. |
| Generalized Anxiety Disorder (GAD-7) | 0.93 | A 7-item anxiety assessment commonly used in primary care. The questions are categorized into none, mild, moderate, and severe anxiety. An increased score is associated with increased anxiety. Previous studies found an internal consistency of 0.89 [9]. |
| Patient Health Questionnaire (PHQ-9) | 0.90 | A 9-item depression module commonly used in primary care. The questions are categorized into none, mild, moderate, moderate-severe, and severe depression. An increased score is corresponding to increased depression. Previous studies found an internal consistency of 0.83-0.92 [10] . |

1. Spielberger, Charles D. 1989. “State-Trait Anxiety Inventory: Bibliography . Palo Alto.” CA: Consulting Psychologists Press.
2. Spielberger, Charles D. 1983. “State-Trait Anxiety Inventory for Adults.” https://doi.org/10.1037/t06496-000.
3. Job, Veronika, Carol S. Dweck, and Gregory M. Walton. 2010. “Ego Depletion—Is It All in Your Head?: Implicit Theories About Willpower Affect Self-Regulation.” *Psychological Science* 21 (11): 1686–93.
4. VanderWeele, Tyler J. 2017. “On the Promotion of Human Flourishing.” *Proceedings of the National Academy of Sciences of the United States of America* 114 (31): 8148–56.
5. Walton, Gregory M., and Geoffrey L. Cohen. 2007. “A Question of Belonging: Race, Social Fit, and Achievement.” *Journal of Personality and Social Psychology* 92 (1): 82–96.
6. Carver, C. S. 1997. “You Want to Measure Coping but Your Protocol’s Too Long: Consider the Brief COPE.” *International Journal of Behavioral Medicine* 4 (1): 92–100.
7. Poulus, Dylan, Tristan J. Coulter, Michael G. Trotter, and Remco Polman. 2020. “Stress and Coping in Esports and the Influence of Mental Toughness.” *Frontiers in Psychology* 11 (April): 628.
8. Kachanoff, Frank, Yochanan Bigman, Kyra Kapsaskis, and Kurt Gray. n.d. “Measuring Realistic and Symbolic Threats of COVID-19 and Their Unique Impacts on Wellbeing and Adherence to Public Health Behaviors.” https://doi.org/10.31234/osf.io/5zr3w.
9. Löwe, Bernd, Oliver Decker, Stefanie Müller, Elmar Brähler, Dieter Schellberg, Wolfgang Herzog, and Philipp Yorck Herzberg. 2008. “Validation and Standardization of the Generalized Anxiety Disorder Screener (GAD-7) in the General Population.” *Medical Care* 46 (3): 266–74.
10. Cameron, Isobel M., John R. Crawford, Kenneth Lawton, and Ian C. Reid. 2008. “Psychometric Comparison of PHQ-9 and HADS for Measuring Depression Severity in Primary Care.” *The British Journal of General Practice: The Journal of the Royal College of General Practitioners* 58 (546): 32–36.
