# Supplementary material for: Risk Factors for COVID-19 in College Students Identified by Physical, Mental, and Social Health Reported During the Fall 2020 Semester: Observational Study Using the Roadmap App and Fitbit Wearable Sensors
Source: JMIR Ment Health. 2022 Feb 10;9(2):e34645. doi: 10.2196/34645 (PMC8834863; doi:10.2196/34645)
Supplement: Multimedia Appendix 6 [file mental_v9i2e34645_app6.doc]

**Multimedia Appendix 6. Correlations of flourishing, compassion, State Trait Anxiety Index (STAI) trait, and public health beliefs.**

| **Correlation** | Flourishing | Compassion | STAI Trait | Public Health Belief |
| --- | --- | --- | --- | --- |
| Flourishing | 1 | **0.224** | **-0.708** | -0.042 |
| Compassion | **0.224** | 1 | **-0.228** | -0.001 |
| STAI Trait | **-0.708** | **-0.228** | 1 | **0.09** |
| Public Health Belief | -0.042 | -0.001 | **0.09** | 1 |

Bold indicates significance at p>0.05
