# Supplementary material for: Risk Factors for COVID-19 in College Students Identified by Physical, Mental, and Social Health Reported During the Fall 2020 Semester: Observational Study Using the Roadmap App and Fitbit Wearable Sensors
Source: JMIR Ment Health. 2022 Feb 10;9(2):e34645. doi: 10.2196/34645 (PMC8834863; doi:10.2196/34645)
Supplement: Multimedia Appendix 7 [file mental_v9i2e34645_app7.doc]

**Multimedia Appendix 7. Odds Ratios of substance use where no report of a mental health condition is the reference group compared to those who reported any mental health condition.**

| Substance | Odds Ratio | 95% CI |
| --- | --- | --- |
| Marijuana | **1.76** | 1.46-2.13 |
| Alcohol | **2.22** | 1.60-2.90 |
| Vaping | **1.64** | 1.32-2.04 |
| Cigarettes | **4.76** | 1.95-11.63 |
