# Supplementary material for: Risk Factors for COVID-19 in College Students Identified by Physical, Mental, and Social Health Reported During the Fall 2020 Semester: Observational Study Using the Roadmap App and Fitbit Wearable Sensors
Source: JMIR Ment Health. 2022 Feb 10;9(2):e34645. doi: 10.2196/34645 (PMC8834863; doi:10.2196/34645)
Supplement: Multimedia Appendix 11 [file mental_v9i2e34645_app11.doc]

**Multimedia Appendix 11. Univariate significant models.**

| Predictor | N | Beta | SE | P-value |  |
| --- | --- | --- | --- | --- | --- |
| Race (Asian) | 1977 | -1.027 | 0.217 | 2.30E-06 | Demographic |
| Race (Multi-racial) | 1977 | -0.993 | 0.467 | 0.03358 |
| International | 1991 | -1.109 | 0.462 | 0.016353 |
| Schoolyear (graduate) | 1994 | -0.828 | 0.324 | 0.010746 |
| Coping: Substance Use | 1986 | 0.429 | 0.095 | 6.20E-06 | Mental |
| Coping: Planning | 1982 | -0.308 | 0.1 | 0.002065 |
| Coping: Humor | 1986 | 0.195 | 0.084 | 0.020465 |
| Coping: Avoidant | 1983 | 0.517 | 0.196 | 0.008349 |
| Marijuana | 1987 | 0.874 | 0.162 | 7.17E-08 | Substance use |
| Alcohol | 1988 | 1.288 | 0.305 | 2.33E-05 |
| Vaping | 1991 | 1.022 | 0.164 | 4.55E-10 |
| Student Social Fit | 1972 | 0.238 | 0.074 | 0.001384 | Other |
| Belief in Public Health | 1967 | -0.289 | 0.055 | 1.43E-07 |
| Loneliness | 1992 | -0.381 | 0.138 | 0.005761 |
| Average daily compliance | 1994 | -0.021 | 0.01 | 0.033631 |
| Belief in getting COVID-19 | 1935 | 1.008 | 0.497 | 0.042631 |
| Housemates (20 +) | 1994 | 1.928 | 0.403 | 1.69E-06 |
| Phone (android) | 1994 | -1.073 | 0.268 | 6.27E-05 |
